# Efficacy and safety of Yangxue Qingnao granules for the treatment of essential hypertension: A protocol for systematic review and meta-analysis

**DOI:** 10.1097/MD.0000000000027911

**Published:** 2021-12-03

**Authors:** Yongcheng Liu, Dong Guo, Ying Tian, Maoxia Fan, Jisen Zhao

**Affiliations:** a College of Traditional Chinese Medicine, Shandong University of Traditional Chinese Medicine, Jinan, China; b Teacher Development Centre, Shandong University of Traditional Chinese Medicine, Jinan, China; c Science and Technology Department, Affiliated Hospital of Shandong University of Traditional Chinese Medicine, Jinan, China; d College of First Clinical Medicine, Shandong University of Traditional Chinese Medicine, Jinan, China.

**Keywords:** essential hypertension, meta-analysis, protocol, systematic review, traditional Chinese medicine, Yangxue Qingnao granules

## Abstract

**Background:**

Essential hypertension is a major risk factor for many fatal cardiovascular and cerebrovascular diseases and has become a heavy burden on families and society. At present, the prevention and treatment of essential hypertension is still unsatisfactory. Yangxue Qingnao granules is a kind of Chinese patent medicine that has been used to treat essential hypertension. The objective of this protocol is to systematically evaluate the efficacy and safety of Yangxue Qingnao granules in the treatment of essential hypertension.

**Methods:**

Randomized controlled trials on Yangxue Qingnao granules for essential hypertension will be searched from the following databases: PubMed, EMbase, Web of Science, Cochrane Library, China National Knowledge Infrastructure, Wanfang Data, China Science and Technology Journal Database, and China Biology Medicine disc from inception to August 27, 2021, regardless of language. Study screening and data extraction will be carried out by two independent reviewers. The quality of the included studies will be assessed using Cochrane risk-of-bias tool for randomized trials. Statistic analysis will be performed using RevMan 5.3 software. The quality of evidence will be assessed using GRADE approach.

**Results:**

This study will systematically evaluate the efficacy and safety of Yangxue Qingnao granules in the treatment of essential hypertension and provide high-quality evidence for clinical practice.

**Conclusion:**

The findings of this systematic review will provide high-quality evidence to verify the efficacy and safety of Yangxue Qingnao granules in the treatment of essential hypertension.

**INPLASY registration number:**

INPLASY202190015.

## 1 Introduction

Essential hypertension is a cardiovascular syndrome with elevated systemic arterial pressure as the main clinical manifestation, accompanied by symptoms such as headache, vertigo and palpitations, ect. In 2010, about 1.39 billion individuals suffered from hypertension globally,^[1]^ and the figure will be close to 1.5 billion by 2025.^[2]^ Stroke, coronary heart disease, end-stage kidney disease and other serious cardiovascular and cerebrovascular complications caused by hypertension lead to high disability and mortality.^[3]^ Worldwide, it is estimated that 10.4 million people die from hypertension each year. As a result, elevated blood pressure remains a leading cause of death and has become a heavy burden on families and society.^[4]^ According to the 2012 to 2015 China Hypertension Survey, the prevalence of hypertension among people over 18 years old in China is 27.9%, showing an increasing trend. However, the awareness, treatment and control rates of hypertension are still at a low level, which are 51.6%, 45.8%, and 16.8% respectively.^[5]^ Thus, it can be concluded that the prevention and treatment of hypertension is still unsatisfactory.

Antihypertensive strategies for essential hypertension mainly include lifestyle adjustment and drug therapy in modern medicine. Common antihypertensive drugs involve calcium channel blockers, angiotensin converting enzyme inhibitors, angiotensin receptor blockers, diuretics and β-blockers, as well as fixed ratio compound formulations composed of the above drugs.^[6]^ Despite the remarkable effect of western antihypertensive drugs, there are still some patients with unsatisfactory blood pressure control. A systematic review based on 24 studies shows that patients with refractory hypertension account for 14% to 16% of the total population with hypertension.^[7,8]^ In addition, with the widespread application of western antihypertensive drugs, disadvantages such as adverse reactions and limited mechanism of action are gradually exposed, leading to poor adherence in patients.^[9,10]^ Therefore, it is of great clinical significance to explore complementary therapies for improving the prevention and control effect of essential hypertension. Traditional Chinese medicine (TCM) can lower blood pressure stably, prevent and cure target organ damage, improve symptoms, and improve patients’ quality of life.^[11]^ As a result, TCM is widely used in the clinical practice of hypertension in China. The combination of Chinese and western drugs can complement each other, lower blood pressure synergistically, improve efficacy and reduce adverse reactions.^[12]^

Yangxue Qingnao granules (YXQNG) used in the treatment of essential hypertension in China is a kind of Chinese patent medicine with the effect of nourishing blood, calming liver, promoting blood circulation and clearing collaterals. It contains components such as phenolic acids, anthraquinones and uncarine, which have the effect of lowering blood pressure and treating cardiovascular diseases.^[13]^ Clinical studies have shown that YXQNG can reduce blood pressure, improve blood pressure variability, improve clinical symptoms such as headache and vertigo, protect target organs, and have fewer adverse reactions.^[14–16]^ Relevant studies suggest that YXQNG may play a therapeutic role in essential hypertension by improving vascular endothelial function, improving hemodynamics, inhibiting the renin-angiotensin-aldosterone system and regulating gene expression.^[13,17]^ However, there is still a lack of high-quality evidence about the efficacy and safety of YXQNG in the treatment of essential hypertension. Therefore, we aim to conduct a systematic review and meta-analysis to verify the efficacy and safety of YXQNG in the treatment of essential hypertension and to provide high-quality evidence for clinical practice.

## 2 Methods

We have registered the protocol on International Platform of Registered Systematic Review and Meta-analysis Protocols (registration number: INPLASY202190015). The protocol is reported strictly according to PRISMA-P.^[18]^

### 2.1 Eligibility criteria

#### 2.1.1 Type of participants

Participants with essential hypertension will be included without limitation of age, gender and race. In accordance with “2020 International Society of Hypertension Global Hypertension Practice Guidelines,”^[6]^ essential hypertension is defined as three measurements of clinic blood pressure on different days with systolic blood pressure (SBP) ≥140mm Hg and/or diastolic blood pressure (DBP) ≥90mm Hg without antihypertensive drugs. Participants with secondary hypertension will be excluded.

#### 2.1.2 Type of interventions and comparators

The control group was given conventional antihypertensive drugs, and the intervention group was given YXQNG on the basis of conventional antihypertensive drugs. Conventional antihypertensive drugs include calcium channel blockers, angiotensin converting enzyme inhibitors, angiotensin receptor blockers, diuretics, β-blockers and fixed ratio compound formulations composed of the above drugs. Basic interventions, if any, should be consistent between the two groups. Studies with less than two weeks of treatment will be excluded.

#### 2.1.3 Type of outcomes

Primary outcomes will be SBP and DBP. Secondary outcomes will include results measured by 24 hours ambulatory blood pressure monitoring (average 24-h SBP, average 24-h DBP, average daytime SBP, average daytime DBP, average nighttime SBP and average nighttime DBP), the effective rate of lowering blood pressure, symptoms, and adverse events.

#### 2.1.4 Type of studies

Randomized controlled trials (RCTs) will be included, regardless of language and publication status. Duplicate publications and studies where complete data cannot be obtained will be excluded.

### 2.2 Information sources and search strategy

#### 2.2.1 Electronic searches

PubMed, EMbase, Web of Science, Cochrane Library, China National Knowledge Infrastructure, Wanfang Data, China Science and Technology Journal Database, and China Biology Medicine disc will be searched by computer from inception to August 27, 2021, regardless of language. A combination of subject words and free words will be used. The retrieval strategy for PubMed is shown in Table 1. Other electronic databases will be searched using the similar retrieval strategy to PubMed.

**Table 1:** Retrieval strategy for PubMed.

#1	Essential Hypertension[MeSH Terms]
#2	Essential Hypertension[Title/Abstract]
#3	Hypertension, Essential[Title/Abstract]
#4	#1 or #2 or #3
#5	Yangxueqingnao[Title/Abstract]
#6	Yangxue Qingnao[Title/Abstract]
#7	Yang Xue Qing Nao[Title/Abstract]
#8	#5 or #6 or #7
#9	Randomized Controlled Trial[Publication Type]
#10	randomized[Title/Abstract]
#11	placebo[Title/Abstract]
#12	#9 or #10 or #11
#13	#4 and #8 and #12

#### 2.2.2 Other searches

Grey literature, Chinese Clinical Trial Registry, and Clinical Trials will also be searched as supplements.

### 2.3 Study selection

**Figure 1:**
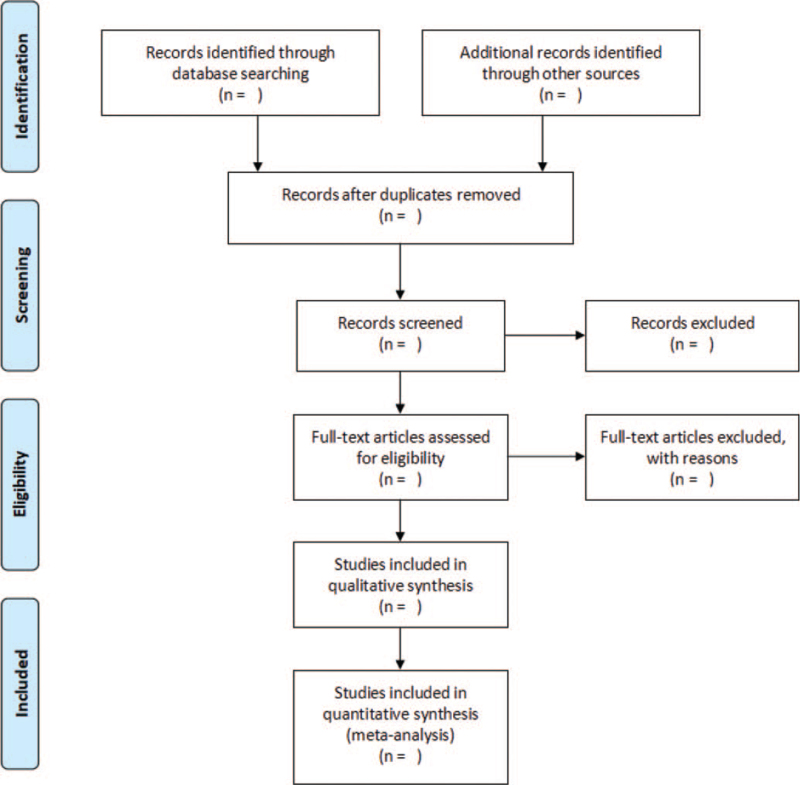
Flow chart of preferred reporting items for systematic review and meta-analysis (PRISMA).

Endnote X9 software will be used to manage the retrieved literature. Two reviewers (YL and MF) will independently screen studies according to eligibility criteria. First, they will read titles and abstracts to exclude duplicate and clearly unqualified studies. Then, they will read the full text of the retained studies after preliminary screening to determine the eligibility. Finally, studies selected will be cross-checked and any disagreement will be resolved by discussion with a third reviewer (YT). The selection process is shown in Figure 1.

### 2.4 Data extraction

Two reviewers (YL and MF) will extract data independently using the pre-established data collection table and finally cross-check. If there is any disagreement, a third reviewer (YT) will be involved. Data extracted from reports will include first author, publication year, title, study design, sample size, age, sex, diagnostic criteria, course of disease, disease grade, complications, interventions, comparators, course of treatment, and outcomes. If the information provided in the literature is incomplete or doubtful, the author will be contacted by telephone or email for relevant information.

### 2.5 Risk of bias assessment

Two reviewers (YL and JZ) will independently evaluate the quality of the included studies using the risk-of-bias tool for randomized trials provided by Cochrane Handbook for Systematic Reviews of Interventions 6.2.^[19]^ The main items include bias arising from the randomization process, bias due to deviations from intended interventions, bias due to missing outcome data, bias in measurement of the outcome and bias in selection of the reported result. Each item is divided into three level: low risk of bias, high risk of bias or some concerns. Any disagreement will be discussed and resolved with a third reviewer (YT).

### 2.6 Statistic analysis

Statistic analysis will be performed using RevMan 5.3 software. For dichotomous data, rate ratio and 95% confidence interval will be used as summary measures. For continuous data, mean difference and 95% confidence interval will be calculated. A *P* value < .05 will be considered significant. Heterogeneity will be explored by Cochrane *Q*-test combined with *I*^*2*^ statistics. When *P* > .1 and *I*^*2*^≤50%, a fixed effect model will be used for meta-analysis. Otherwise, heterogeneity will be considered significant and a random effect model will be used when *P* < .1 or *I*^*2*^ > 50%. Sensitivity analysis and subgroup analysis will be conducted to find the cause of significant heterogeneity. If meta analysis is not appropriate, we will only perform a descriptive analysis.

### 2.7 Subgroup analysis

If there is significant heterogeneity in included studies, subgroup analysis will be conducted according to age, hypertension grade, dosage of YXQNG, type of conventional antihypertensive drugs, course of treatment, and TCM syndrome type.

### 2.8 Sensitivity analysis

To ensure the stability of the synthetical results, sensitivity analysis will be carried out by eliminating individual studies one by one.

### 2.9 Publication bias

When 10 or more studies are included in the meta-analysis, the funnel plot will be used to assess publication bias. The asymmetric funnel plot indicates the possibility of publication bias.

### 2.10 Evidence quality assessment

The GRADE approach will be used to assess the quality of evidence which will be graded as high, moderate, low and very low. Evidence can be degraded by limitations, inconsistency, indirectness, imprecision or publication bias, and also can be upgraded by large effect, plausible confounding that would change the effect and dose-response gradient.

### 2.11 Ethics and dissemination

Our research collects published studies and there is no personal privacy information included. Thus, ethical approval is not required. Results of this systematic review will be published in a peer-reviewed journal.

## 3 Discussion

Hypertension has become an important international public health problem in the context of the continuous increase in the number of hypertensive patients worldwide. Despite the fact that many proven, highly effective lifestyle and drug treatment strategies can lower blood pressure, hypertension control rate remains unsatisfactory.^[20]^ Therefore, it is of great significance to explore new coping strategies for the prevention and treatment of hypertension. In TCM, diseases are named after symptoms, and essential hypertension belongs to the category of headache and vertigo. TCM believes that the causes of hypertension mainly include improper diet, emotional disorders, lose balance between work and rest, and physical deficiency caused by old age or long illness. The pathogeneses are deficiency of liver and kidney Yin, hyperactivity of liver Yang, and stagnation of blood stasis and phlegm.^[12]^ YXQNG used in the treatment of essential hypertension in China has the effect of nourishing Yin blood, calming liver Yang, activating blood circulation and dredging the meridian. Clinical studies have proved that YXQNG can reduce blood pressure, improve the accompanying symptoms,^[16]^ and prevent target organs damage.^[15]^ However, the lack of high-quality evidence limits the wide application of YXQNG. Therefore, we expect to confirm the efficacy and safety of YXQNG in the treatment of essential hypertension through this systematic review and meta-analysis, so as to provide high-quality evidence for clinical decision-making. However, this study also has some potential limitations. On the one hand, there are fewer RCTs with multi-center, large sample and high quality. On the other hand, differences in age, complications and types of conventional antihypertensive drugs may cause significant heterogeneity. Consequently, more high-quality RCTs should be carried out in the future to verify the efficacy and safety of YXQNG in the treatment of essential hypertension.

## Author contributions

**Conceptualization:** Yongcheng Liu, Ying Tian, Dong Guo.

**Data curation:** Yongcheng Liu, Ying Tian, Maoxia Fan, Jisen Zhao.

**Formal analysis:** Yongcheng Liu, Ying Tian.

**Methodology:** Ying Tian.

**Software:** Yongcheng Liu, Maoxia Fan, Jisen Zhao.

**Supervision:** Dong Guo.

**Writing – original draft:** Yongcheng Liu.

**Writing – review & editing:** Dong Guo.
